# Geometry-Informed Adaptive Time-Series Fusion of ADS-B Sensor Data for Short-Term Aircraft Trajectory Prediction

**DOI:** 10.3390/s26165091

**Published:** 2026-08-11

**Authors:** Yunfeng Wan, Xinyu Zhao, Benkui Zhang, Lei Dai, Yichang Luo, Mingli Xie, Ying Chang

**Affiliations:** 1 Aerospace Information Research Institute, Chinese Academy of Sciences, Beijing 100190, China; wanyunfeng24@mails.ucas.ac.cn (Y.W.); zhaoxinyu@aircas.ac.cn (X.Z.); zhangbk@aircas.ac.cn (B.Z.); luoyichang24@mails.ucas.ac.cn (Y.L.); xiemingli24@mails.ucas.ac.cn (M.X.); 2 Key Laboratory of Target Cognition and Application Technology (TCAT), Beijing 100190, China; 3 University of Chinese Academy of Sciences , Beijing 100049, China; 4 School of Electronic, Electrical and Communication Engineering, University of Chinese Academy of Sciences, Beijing 100049, China; 5 School of Artificial Intelligence, Beijing University of Posts and Telecommunications (BUPT), Beijing 100876, China; dailei2024@bupt.edu.cn

**Keywords:** ADS-B sensor data, aircraft trajectory prediction, aviation surveillance, geometric–kinematic representation, time-series forecasting, adaptive fusion

## Abstract

Automatic Dependent Surveillance-Broadcast (ADS-B) systems provide continuous aircraft position reports for aviation surveillance and short-term trajectory prediction. However, many data-driven predictors directly model the longitude, latitude, and altitude contained in ADS-B messages as generic multivariate time-series variables, which can weaken latitude-dependent displacement, bearing, and local motion relationships during multi-step forecasting. This paper proposes Geometry-Informed Adaptive Time-Series Fusion (GeoATF), a forecasting framework for short-term aircraft trajectory prediction from ADS-B position reports. For multi-route learning, each input window is mapped from global absolute coordinates to a local geodesic chart anchored at the last observation. The predicted local offsets are subsequently decoded into World Geodetic System 1984 (WGS-84) coordinates through great-circle forward navigation. GeoATF combines a patch-based time-series Transformer (PatchTST) temporal branch for modeling historical ADS-B position sequences with a geometric–kinematic branch that constructs great-circle distance, bearing, velocity component, and trajectory change rate features from the same sequence. An adaptive branch weight fusion module then learns horizon-dependent geometric–kinematic branch weights for residual correction. Experiments on 945 ADS-B flights from ten routes demonstrate that GeoATF delivers more accurate multi-horizon trajectory forecasts than the evaluated baselines, with more pronounced advantages at longer forecast horizons. Flight-level statistical analysis supports the robustness of these improvements, while resource evaluation indicates a moderate computational footprint under the common inference protocol. These results suggest that explicitly representing geometric–kinematic information and adaptively fusing it with temporal features can improve short-term aircraft trajectory prediction using only historical ADS-B position data.

## 1. Introduction

Trajectory-based operations and aviation surveillance require reliable forecasts of near-future aircraft motion from continuously reported position observations. Automatic Dependent Surveillance-Broadcast (ADS-B) provides low-cost, high-update-rate aircraft position reports and supports traffic monitoring, conflict risk awareness, arrival flow prediction, and trajectory-based decision making [1,2,3]. The usefulness of these reports for prediction depends not only on the forecasting architecture, but also on whether longitude, latitude, and altitude are represented in a physically meaningful form.

Model-driven and filtering-based predictors provide interpretable short-term state estimates, but they commonly require motion assumptions, intent information, or aircraft-specific parameters that may be unavailable in heterogeneous open ADS-B records [4,5,6]. Data-driven methods reduce this dependence by learning directly from historical trajectories [7,8,9,10,11]. Nevertheless, many such models treat World Geodetic System 1984 (WGS-84) longitude and latitude as generic Euclidean variables, although the physical distance represented by a longitude increment varies with latitude and horizontal motion is naturally described by geodesic distance and bearing. This representation mismatch can introduce spatial errors that accumulate during multi-step forecasting.

The methodological question considered in this study is therefore how to preserve the flexibility of a modern time-series forecaster while making the coordinate representation and residual correction explicitly geometry-aware. The proposed Geometry-Informed Adaptive Time-Series Fusion (GeoATF) framework addresses this question using only historical ADS-B longitude, latitude, and altitude; it does not assume access to flight plans, weather, controller instructions, or proprietary aircraft intent variables.

The work is organized as follows. Section 2 reviews the state of the art and formulates the research aim and tasks. Section 3 presents the proposed method. Section 4 describes data acquisition and preprocessing. Section 5 defines the experimental protocol. Section 6 reports the results. Section 7 discusses their interpretation and limitations, and Section 8 concludes the paper.

## 2. State of the Art

### 2.1. ADS-B-Based Flight Trajectory Prediction

Flight trajectory prediction is commonly formulated as a multivariate time-series forecasting problem in which future aircraft positions are inferred from historical aircraft position observations. ADS-B data are well suited to this task because they provide rich position and flight-status records from aircraft broadcast messages. Previous studies have explored ADS-B-based trajectory prediction with hybrid deep learning models [1], interacting-multiple-model and Informer fusion [12], convolutional neural network–long short-term memory (CNN–LSTM) structures [7], and Bayesian uncertainty-aware neural networks [13]. In aviation surveillance and sensing applications, aircraft trajectory and ADS-B-related information have also been used for air/ground surveillance data processing, satellite-based aircraft detection, and ADS-B signal analysis [14,15,16]. Other studies seek to improve prediction by incorporating weather uncertainty, terminal area information, phase-aware modeling, or time-frequency representations [8,17,18,19].

Before recent deep-learning architectures became dominant, ADS-B and flight plan trajectory prediction were also studied with predictive analytics, relative-motion features, hidden Markov models, support vector regression, decision trees, and generative convolutional-recurrent structures [20,21,22,23,24]. These studies are relevant to the present work because they show that trajectory prediction benefits from domain-specific representations rather than relying only on generic sequence regression. They also reveal a persistent difficulty: Handcrafted motion assumptions are interpretable, but they may not adapt well to heterogeneous aircraft behavior, while purely data-driven representations can fit historical patterns but may ignore the geometric structure of geodetic coordinates.

Prediction horizons are often categorized as short-term, medium-term, and long-term according to the operational use case [13,25,26]. Long-term methods often emphasize route-level evolution, intent, or goal-oriented generation [27,28], whereas recent short-term methods focus on local temporal dynamics and multi-scale motion patterns [29]. The present study focuses on short-term, multi-step prediction, with the maximum horizon equal to 16 samples at a 15 s sampling interval, i.e., 240 s. This range is relevant for near-term trajectory monitoring and data-driven surveillance applications, but it should not be confused with strategic long-term trajectory prediction for flow management or demand capacity planning.

### 2.2. Model-Driven and Filtering-Based Methods

Model-driven approaches describe aircraft motion through physical or kinematic equations. Point mass models, total energy models, and aircraft performance databases such as the Base of Aircraft Data (BADA) have been used for trajectory prediction and four-dimensional (4D) trajectory optimization [30,31,32,33]. Classical automated en-route trajectory modeling and later trajectory-based operations (TBO) studies further connect prediction accuracy with downstream air traffic functions [2,34]. These approaches are interpretable and physically meaningful, but they often depend on accurate aircraft-specific parameters, environmental conditions, and intent information. Recent work has used machine learning to estimate aircraft performance model parameters [6], which suggests that the boundary between model-driven and data-driven prediction is increasingly hybrid rather than strictly separated.

Filtering-based and probabilistic methods estimate aircraft states recursively from noisy measurements. Kalman filtering and its nonlinear variants are widely used for tracking and short-term prediction [4,35,36], while probabilistic trajectory prediction has also been studied for conflict-related applications [37,38]. For maneuvering targets, multiple model estimators can represent different motion hypotheses and combine their state estimates [5]. Related sensor fusion and aircraft-monitoring studies further show that surveillance data fusion, ADS-B crowd-sensor networks, and optical/infrared and synthetic aperture radar (SAR)-based aircraft trajectory recognition can support robust tracking, anomaly detection, or airborne target analysis [39,40,41]. These methods are closely related to sensor fusion because they explicitly model observation uncertainty and state evolution. However, their performance depends on the selected state-space models and noise assumptions. In heterogeneous ADS-B trajectories, a single fixed motion model may be insufficient for changing flight phases and complex maneuvers, while detailed aircraft intent or operational constraints are often unavailable in open ADS-B data.

### 2.3. Deep Learning and Trajectory Representation

Data-driven methods learn trajectory evolution from historical records without explicitly specifying the complete aircraft dynamics. Recurrent neural networks, LSTM, CNN–LSTM, Transformer-based methods, and binary-encoding models have been applied to flight trajectory prediction [7,9,10,42,43]. Constrained LSTM, intent-derived terminal-area prediction, and wavelet-based representations further suggest that domain information and phase-dependent patterns can improve prediction robustness [8,44,45]. The patch-based time-series Transformer (PatchTST) [46] and related Transformer variants have also shown competitive performance in time-series forecasting by modeling temporal dependencies through patching and attention [47].

Representation learning has become an important theme in trajectory modeling. Graph-based, hierarchical, hypergraph, and domain-adaptive representations have been used in transportation, vessel, multi-agent, and motion prediction tasks [48,49,50,51,52,53]. In aviation, the global and local interattribute relationships-based graph convolutional network (GLAR-GCN) constructs global and local interattribute graphs for flight trajectory prediction [11], FlightBERT and FlightBERT++ encode trajectory states into structured binary representations [9,10], and multi-scale modeling has recently been explored for short-term flight prediction [29]. These studies indicate that prediction quality depends not only on the forecasting backbone but also on how trajectory observations are represented. The present work follows this representation learning direction while remaining self-contained with respect to input data: All added information is derived from the observed ADS-B position sequence through standard geometric and kinematic transformations.

The quality of positioning time series is also relevant to data-driven trajectory modeling. Long global navigation satellite system (GNSS) coordinate records exhibit repeatable seasonal variations and component-dependent fitting dispersion [54]; gaps in GNSS position time series require explicit interpolation and error assessment [55]; and self-attention-based prediction of GNSS coordinate series uses synchronized sliding-window preprocessing [56]. Although these studies address geodetic stations rather than aircraft, they reinforce the need to document positioning-data quality control, temporal sampling, missing-data handling, and coordinate representation when constructing an ADS-B forecasting benchmark.

### 2.4. Research Gaps and Objectives

The state of the art reveals two connected gaps. First, generic sequence models do not explicitly preserve the geodesic meaning of WGS-84 coordinates when trajectories from geographically separated routes are learned jointly. Second, geometry-derived motion cues are often concatenated or applied with a fixed contribution, although their usefulness can vary with prediction horizon and flight phase. The aim of this study is to determine whether an anchor-relative geodesic representation, a geometric–kinematic branch, and adaptive branch-weight fusion can improve short-term multi-step ADS-B trajectory prediction without external operational variables.

To achieve this aim, the study pursues four objectives: (i) to construct a chronologically partitioned, multi-route ADS-B benchmark and document its acquisition and quality control procedure; (ii) to formulate a last-observation-anchored local geodesic prediction representation and derive geometric–kinematic descriptors from the observed positions; (iii) to combine these descriptors with a patch-based temporal forecaster through horizon-dependent adaptive fusion; and (iv) to evaluate predictive accuracy, paired flight-level significance, sampling interval sensitivity, module ablations, flight-phase behavior, qualitative trajectories, and computational resource demand under a reproducible protocol.

## 3. Proposed Method

### 3.1. Problem Definition

Let an ADS-B trajectory be represented as a sequence of geographic observations:

(1) $$X_{1:T}={\left[x_{1},x_{2},\ldots ,x_{T}\right]}^{⊤}\in R^{T\times 3},\;x_{t}={\left[\lambda_{t},\phi_{t},h_{t}\right]}^{⊤},$$

where $\lambda_{t}$, $\phi_{t}$, and $h_{t}$ denote longitude, latitude, and altitude at time step *t*, respectively. The longitude and latitude channels are treated as WGS-84 geodetic coordinates, and altitude is used as the vertical coordinate. WGS-84 coordinates remain the external input/output and reporting system; inside GeoATF, each window is mapped to a local geodesic chart anchored at its final observed position, as defined below. Given a historical window of length *T*, the prediction task is to estimate the future trajectory segment:

(2) $${\hat{Y}}_{T+1:T+H}=f_{\theta }\left(X_{1:T}\right),\;{\hat{Y}}_{T+1:T+H}\in R^{H\times 3},$$

where *H* is the prediction horizon and $f_{\theta }\left(\cdot \right)$ is the learnable prediction model.

This formulation uses only the position-related fields that are consistently available in the processed ADS-B records. Although additional surveillance fields such as ground speed, vertical rate, heading, or operational intent can be useful when reliably available, the present setting intentionally focuses on the minimum coordinate sequence. This makes the evaluation stricter; the model must infer short-term motion tendency from the observed coordinate evolution itself. It also motivates the explicit construction of geometric–kinematic features in the following sections, because raw coordinate values have different physical meanings along different axes and at different latitudes.

### 3.2. Last-Observation-Anchored Local Geodesic Representation

Directly regressing globally standardized absolute coordinates is poorly conditioned for a model trained jointly on geographically separated routes because route-dependent coordinate offsets dominate the numerical representation. We therefore define a local geodesic chart for every input window. Let the anchor be the final observed point $a=x_{T}=\left(\lambda_{T},\phi_{T},h_{T}\right)$. For any point $x_{t}$, let $d(a,x_{t})$ and $\psi (a,x_{t})$ denote its spherical great-circle distance and initial bearing from the anchor, with the longitude difference wrapped to [−π,π]. The local state is

(3) $$r_{t}=\Gamma_{a}\left(x_{t}\right)={\left[\frac{(h_{t}-h_{T})/1000}{s_{U}},\frac{d\left(a,x_{t}\right)\sin\psi \left(a,x_{t}\right)}{s_{H}},\frac{d\left(a,x_{t}\right)\cos\psi \left(a,x_{t}\right)}{s_{H}}\right]}^{⊤},$$

where the components are normalized up, east, and north offsets, respectively, and $s_{U}=10$ km and $s_{H}=100$ km are fixed scale factors. Both prediction branches operate on this anchor-relative representation. Their future local outputs are converted back to WGS-84 longitude, latitude, and altitude by the standard great-circle forward-navigation map $\Gamma_{a}^{-1}$. The spherical map uses the mean Earth radius R=6371.0088 km, while the training and evaluation distance described later uses the WGS-84 ellipsoid. Thus, the revision replaces route-dependent global absolute-coordinate regression with translation-independent local geodesic prediction without introducing a constant-velocity reference or any external variable.

### 3.3. Overall Framework

**Figure 1:**
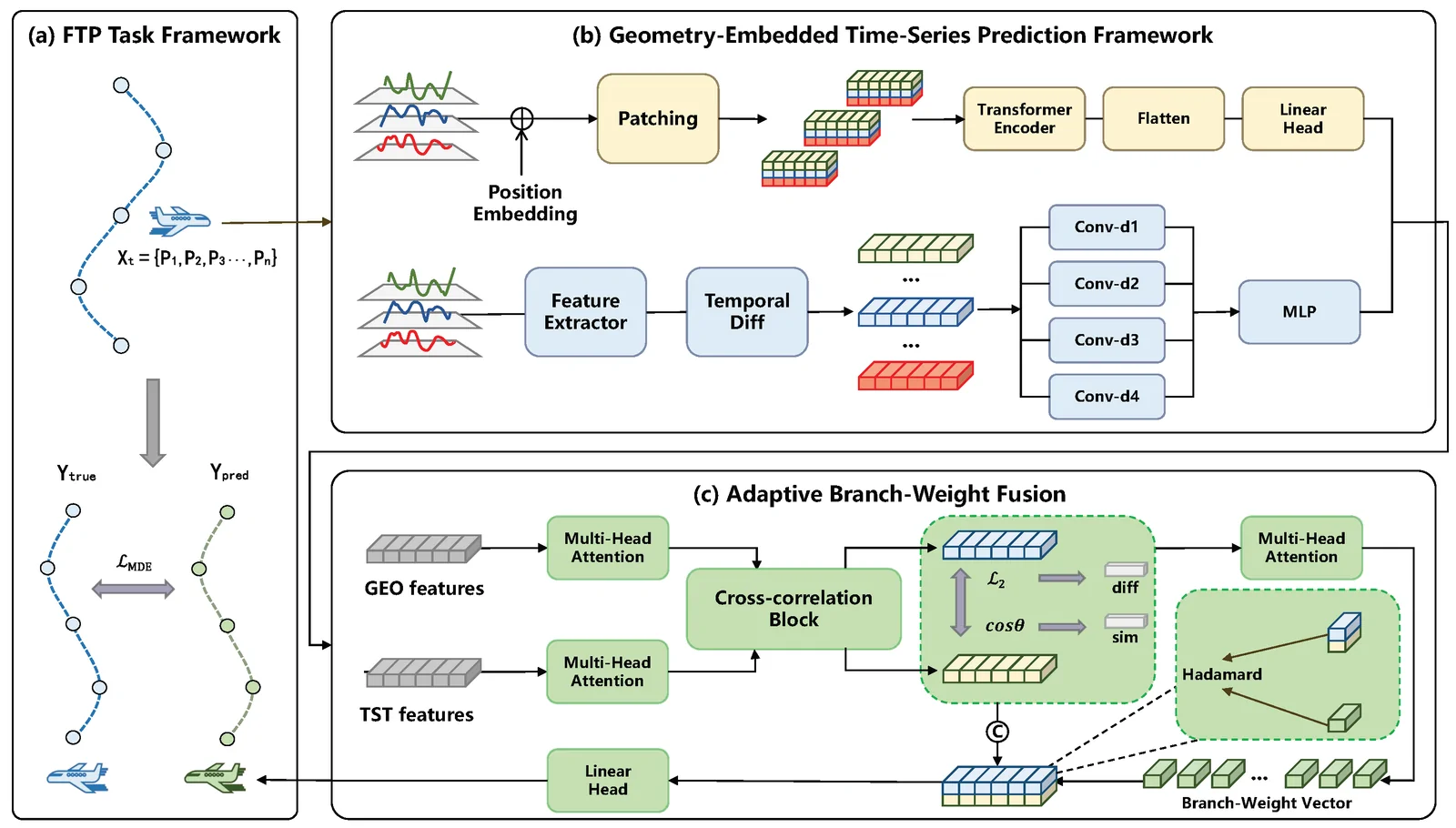
Architecture of Geometry-Informed Adaptive Time-Series Fusion (GeoATF). Panel (**a**) presents the forecasting task; panel (**b**) shows the temporal branch (beige) and geometric–kinematic branch (blue); and panel (**c**) illustrates adaptive branch-weight fusion (green). The multicolored traces and tensors in panel (**b**) depict the three coordinate channels. Solid arrows indicate forward information flow, double-headed arrows indicate pairwise feature comparison, dashed connectors denote auxiliary inputs to branch-weighted fusion, and the circled *C* and Hadamard labels denote concatenation and element-wise multiplication, respectively.

The proposed GeoATF framework combines a temporal branch, a geometric–kinematic branch, and an adaptive fusion module. Figure 1 illustrates the overall structure. The design follows a simple principle: The temporal branch learns sequence regularities from the coordinate history, while the geometric–kinematic branch converts the same history into physically meaningful motion cues before temporal abstraction. The fusion module then learns how much geometry-informed correction should be applied to the prediction.

In the upper branch of Figure 1, the local history $R_{1:T}=\Gamma_{a}\left(X_{1:T}\right)$ is processed as a multivariate time series. This branch preserves the data-driven forecasting ability of a modern time-series backbone and does not impose a constant-velocity reference. In parallel, the geometric–kinematic branch combines the same local history with motion quantities calculated from the physical WGS-84 observations. The two-branch representation is defined as

(4) $$\begin{array}{c} F_{T}=E_{T}\left(R_{1:T};\theta_{T}\right),\\ {\tilde{Z}}_{1:T}=\Phi_{\mathrm{GK}}\left(R_{1:T},X_{1:T}\right),\\ F_{G}=E_{G}\left({\tilde{Z}}_{1:T};\theta_{G}\right), \end{array}$$

where $E_{T}\left(\cdot \right)$ is implemented with a patch-based Transformer structure, $\Phi_{\mathrm{GK}}\left(\cdot \right)$ denotes deterministic geometric–kinematic feature construction, and $E_{G}\left(\cdot \right)$ is the multi-scale convolutional encoder. The lower branch in Figure 1 is therefore not an independent data source, but a transformed view of the same ADS-B position sequence. Let ${\hat{R}}^{\left(T\right)}=\Pi_{T}\left(F_{T}\right)$ and ${\hat{R}}^{\left(G\right)}=\Pi_{G}\left(F_{G}\right)$ be the temporal and geometric–kinematic predictions in the local chart. The final prediction is

(5) $$\begin{array}{c} \hat{R}={\hat{R}}^{\left(T\right)}+\beta \;G⊙\left({\hat{R}}^{\left(G\right)}-{\hat{R}}^{\left(T\right)}\right),\\ {\hat{Y}}_{T+1:T+H}=\Gamma_{a}^{-1}\left(\hat{R}\right), \end{array}$$

where $G\in {\left[0.1,0.9\right]}^{H}$ is the learned horizon-dependent geometric branch weight, ⊙ denotes the Hadamard product, and the direct-branch scale is fixed at β=1 in the reported experiments.

The residual form in Equation (5) is used to keep the temporal prediction as the main forecasting path while allowing the geometric–kinematic branch to correct accumulated spatial deviations. This is different from simply concatenating all features before prediction. Direct concatenation treats geometric, kinematic, and temporal features as equally reliable at all horizons, whereas the proposed fusion module allows the contribution of the geometry-informed component to vary with feature consistency and prediction step.

### 3.4. Geometric–Kinematic Feature Construction

**Figure 2:**
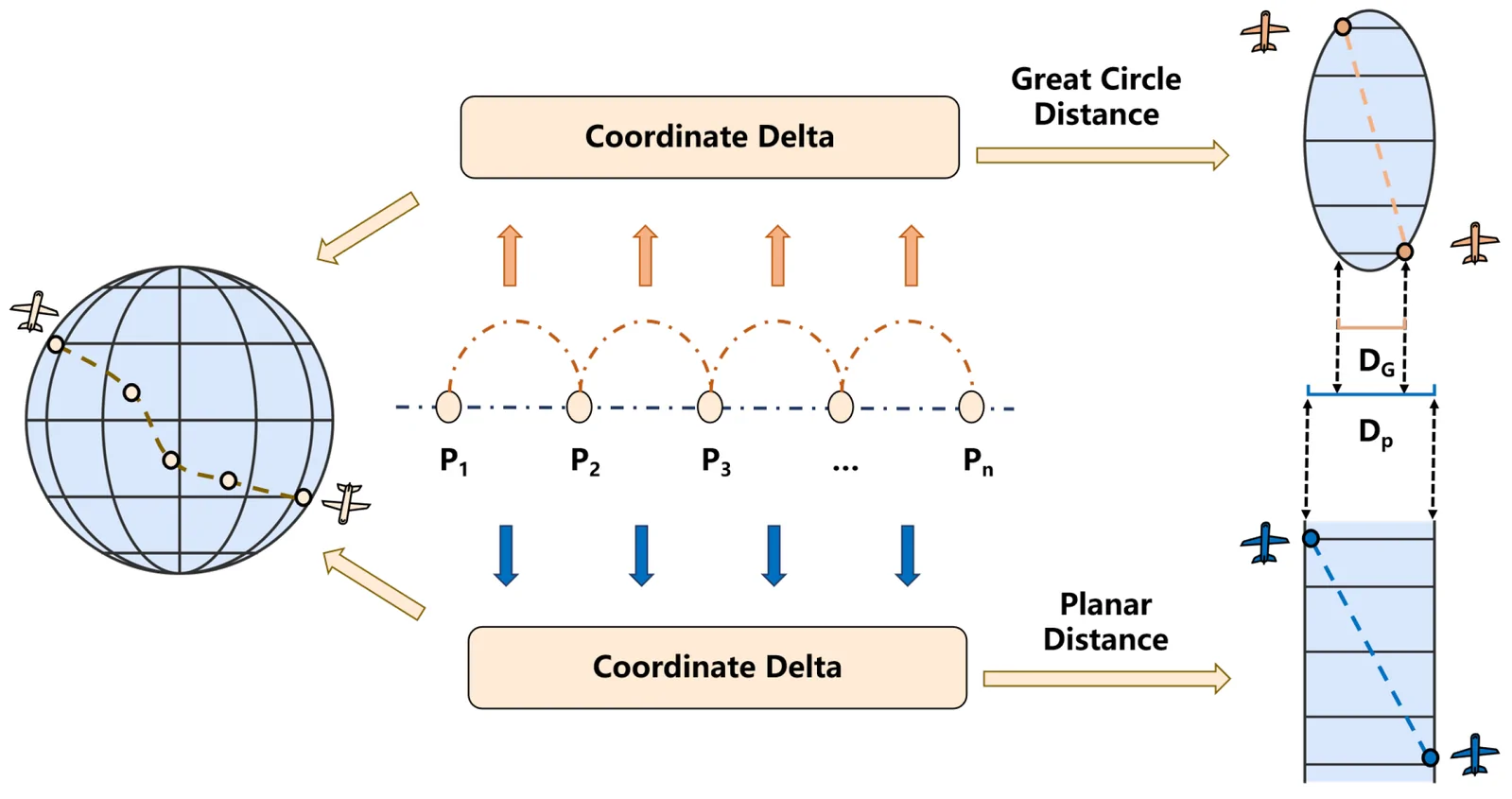
Latitude-dependent longitude-to-distance scaling. Orange and blue arrows denote the great-circle distance $D_{G}$ and planar distance $D_{P}$, respectively.

The geometric–kinematic feature extraction (GKFE) module supplies the learning model with geometry-informed motion descriptors that are not explicit in a raw longitude–latitude–altitude sequence. The key issue is that longitude and latitude are WGS-84 geodetic coordinates rather than planar Cartesian coordinates. If a model learns directly from numerical coordinate differences, it implicitly treats longitude and latitude as if they were planar Cartesian variables. This approximation ignores the latitude-dependent scale of longitude and the spherical geometry of horizontal displacement; for the same longitude increment, the corresponding physical distance changes with latitude, and the representation bias can become more pronounced over routes with latitude variation or over accumulated multi-step predictions. Therefore, instead of using raw coordinate differences as the only motion cues, the module derives great-circle distance, bearing, and velocity-related descriptors from consecutive WGS-84 positions and uses them as auxiliary inputs to the forecasting model. The learnable position channels are the local states from Equation (3), and the predicted local states are decoded to WGS-84 coordinates only after fusion. Figure 2 illustrates the motivation that longitude-distance scaling varies with latitude.

For two consecutive geographic positions $P_{t-1}=\left(\lambda_{t-1},\phi_{t-1},h_{t-1}\right)$ and $P_{t}=\left(\lambda_{t},\phi_{t},h_{t}\right)$, standard spherical navigation operators are used to obtain the great-circle distance $d_{t}$ and the initial bearing $\psi_{t}$. These quantities provide physically interpretable horizontal-motion cues for the subsequent temporal encoder. The resulting local horizontal motion descriptor is

(6) $$c_{t}={\left[\frac{d_{t}\sin\psi_{t}}{\Delta t},\frac{d_{t}\cos\psi_{t}}{\Delta t},\psi_{t}\right]}^{⊤},$$

where the first two components correspond to eastward and northward velocity terms in meters per second. The curvature-aware descriptor is written as $x_{t}^{\left(6\right)}={\left[r_{t}^{⊤},c_{t}^{⊤}\right]}^{⊤}$. For the first point in each sliced window, which has no predecessor inside that window, the first observable motion is repeated instead of inserting an artificial zero-velocity state.

To describe recent trajectory variation, the module further augments this descriptor with finite-difference motion terms:

(7) $${\tilde{z}}_{t}={\left[x_{t}^{\left(6\right)},\Delta x_{t}^{\left(6\right)},\Delta^{2}x_{t}^{\left(6\right)}\right]}^{⊤}.$$

Here, $\Delta x_{t}^{\left(6\right)}$ and $\Delta^{2}x_{t}^{\left(6\right)}$ denote first- and second-order temporal change descriptors computed from recent observations. Their first boundary values repeat the first computable difference, avoiding a discontinuity created solely by window slicing. Thus, ${\tilde{z}}_{t}$ combines anchor-relative position, latitude-aware horizontal motion, bearing, and local trajectory change information without introducing additional external data.

Multi-scale temporal features are extracted from the geometric–kinematic sequence using parallel dilated convolution blocks:

(8) $$\begin{array}{c} H_{d}=\sigma \left(\mathrm{BN}\left({\mathrm{Conv}}_{\kappa ,d}\left({\tilde{Z}}_{1:T}\right)\right)\right),\;d\in D,\\ F_{G}=\Pi_{G}\left(\mathrm{Concat}\left({\left\{H_{d}\right\}}_{d\in D}\right)\right),\;D=\left\{1,2,4,8\right\}. \end{array}$$

Here, ${\mathrm{Conv}}_{\kappa ,d}$ denotes a one-dimensional convolution with kernel size κ and dilation *d*, and BN denotes batch normalization. Compared with directly feeding longitude, latitude, and altitude into a sequence model, this representation exposes the model to physically meaningful displacement and turning information before temporal abstraction. The different dilation rates allow the branch to describe both immediate motion changes and smoother local trends within the same historical window. In this way, the geometric–kinematic branch complements the patch-based temporal branch rather than duplicating it.

### 3.5. Adaptive Branch-Weight Fusion

The adaptive branch-weight fusion (ABF) module combines temporal patterns and geometric–kinematic features according to a learned branch-weighting signal. Bidirectional cross-branch interaction is first used to exchange information between the temporal and geometric–kinematic branches. For two generic feature sequences U and V, the scaled dot-product attention used in the interaction block is

(9) $$\mathrm{Attn}\left(U,V\right)=\mathrm{softmax}\left(\frac{\left(UW_{Q}\right){\left(VW_{K}\right)}^{⊤}}{\sqrt{d_{k}}}\right)VW_{V}.$$

Using this operator, bidirectional branch interaction and residual normalization are written as

(10) $$\begin{array}{c} A_{T\leftarrow G}=\mathrm{Attn}\left(F_{T},F_{G}\right),\\ A_{G\leftarrow T}=\mathrm{Attn}\left(F_{G},F_{T}\right),\\ {\tilde{F}}_{T}=\mathrm{LN}\left(F_{T}+\alpha_{T}A_{T\leftarrow G}\right),\\ {\tilde{F}}_{G}=\mathrm{LN}\left(F_{G}+\alpha_{G}A_{G\leftarrow T}\right). \end{array}$$

**Figure 3:**
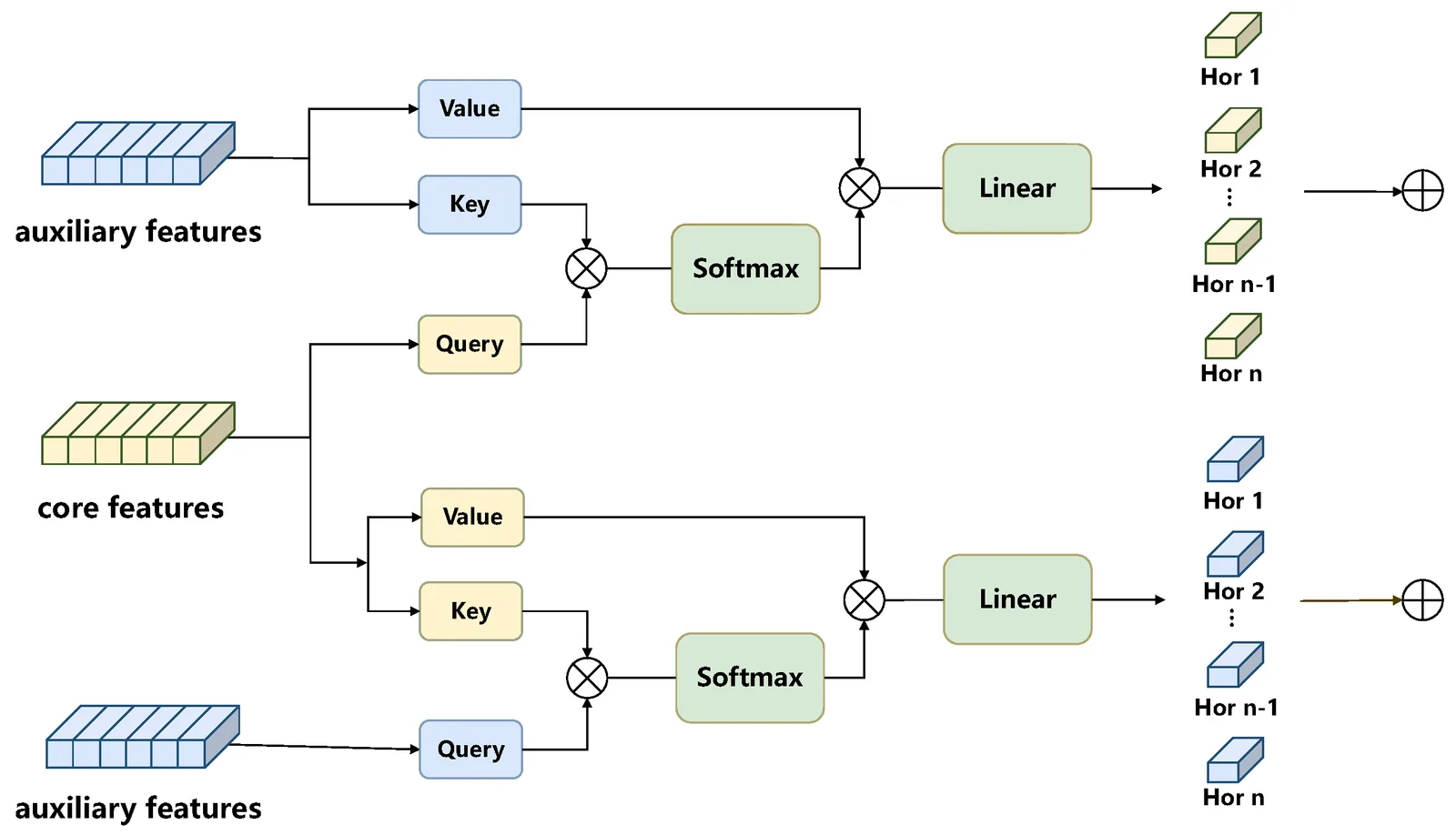
Bidirectional cross-correlation block. Blue and beige denote auxiliary and core tensors; arrows show information flow.

Here, $W_{Q}$, $W_{K}$, and $W_{V}$ are learnable projection matrices, $d_{k}$ is the key dimension, LN(·) denotes layer normalization, and $\alpha_{T}$ and $\alpha_{G}$ are learnable interaction coefficients. Figure 3 illustrates this interaction block.

The cross-branch interaction is introduced before branch-weight estimation so that each branch can access complementary information from the other branch. If both branches encode similar motion tendencies, the fusion module can assign a larger weight to the geometric–kinematic residual. If their representations diverge, the gate can reduce the correction and rely more strongly on the temporal forecasting path. For every forecast step *h*, the normalized calibrated branch features are first compared through a scalar cosine-similarity descriptor:

(11) $$s_{h}=\frac{\langle {\tilde{f}}_{T,h},{\tilde{f}}_{G,h}\rangle }{{∥{\tilde{f}}_{T,h}∥}_{2}{∥{\tilde{f}}_{G,h}∥}_{2}+\epsilon }.$$

Their feature-space disagreement is summarized by an $\ell_{2}$ distance:

(12) $$\delta_{h}={∥{\tilde{f}}_{T,h}-{\tilde{f}}_{G,h}∥}_{2}.$$

Together with a prediction-horizon encoding $e_{h}$, these descriptors form the horizon-aware branch-consistency descriptor:

(13) $$q_{h}=\left[{\tilde{f}}_{T,h},{\tilde{f}}_{G,h},s_{h},\delta_{h},e_{h}\right].$$

A multilayer perceptron (MLP) analyzes $q_{h}$, and self-attention across the *H* analyzed descriptors permits horizon-to-horizon calibration. If ${\bar{u}}_{h}$ denotes the attended descriptor, the implemented branch weight is

(14) $${\tilde{G}}_{h}=\sigma \left(w_{2}^{⊤}{\bar{u}}_{h}+b_{2}\right),\;G_{h}={\mathrm{clip}}_{\left[0.1,0.9\right]}\left(a{\tilde{G}}_{h}+b_{h}\right).$$

Finally, G modulates the branch residual in Equation (5). In Equations (11)–(14), ϵ is a small constant for numerical stability, σ(·) is the sigmoid function, *a* is a learned global scale, and $b_{h}$ is a learned horizon-specific bias. Clipping prevents either branch from being assigned a numerically degenerate zero or unit weight. The gate is a learned branch weight rather than a calibrated probabilistic uncertainty estimate. This design adapts the geometric–kinematic correction to branch consistency and prediction horizon.

The model is trained with the same physical quantity used by the primary spatial evaluation, the three-dimensional mean distance error (MDE). Let $d_{E}\left(\cdot ,\cdot \right)$ be the Euclidean distance after the differentiable WGS-84 geodetic-to-Earth-centered, Earth-fixed (ECEF) mapping in Equation (17). The multi-step objective over a mini-batch B is

(15) $$\begin{array}{c} L_{\mathrm{total}}\left(\Theta \right)=L_{\mathrm{MDE}}\left(\hat{Y},Y\right)+\gamma \;L_{\mathrm{MDE}}\left({\hat{Y}}^{\left(G\right)},Y\right),\\ L_{\mathrm{MDE}}=\frac{1}{|B|H}\sum_{i\in B}\sum_{h=1}^{H}d_{E}\left({\hat{y}}_{i,T+h},y_{i,T+h}\right), \end{array}$$

where Θ denotes all learnable parameters, ${\hat{Y}}^{\left(G\right)}$ is the decoded geometry-branch prediction, and γ=0.1 provides direct auxiliary supervision to that branch. The target and both predictions are de-standardized before the ECEF transform.

For clarity, Algorithm 1 summarizes the complete prediction procedure. The algorithm is written at the model-operation level rather than at the implementation level, because the deterministic geometric–kinematic feature construction and the learnable fusion module are the two key components that distinguish the proposed framework from a standard time-series forecaster.


**Algorithm 1** (GeoATF geometry-informed adaptive trajectory prediction procedure).
**No.**

**Stage**

**Operation**
InputHistorical ADS-B sequence $X_{1:T}={\left[x_{1},\ldots ,x_{T}\right]}^{⊤}$ with $x_{t}=\left(\lambda_{t},\varphi_{t},h_{t}\right)$; horizon *H*; learnable parameters Θ.OutputPredicted trajectory ${\hat{Y}}_{T+1:T+H}$.1Local geodesic chartAnchor the window at $a=x_{T}$ and map every observation to normalized up–east–north coordinates
$R_{1:T}=\Gamma_{a}\left(X_{1:T}\right)$ using great-circle inverse navigation.2State liftingConstruct physical geometric–kinematic descriptors from consecutive WGS-84 observations:
$d_{t}$ and $\psi_{t}$ by standard spherical navigation operators,
$v_{t}^{E}=d_{t}\sin\psi_{t}/\Delta t$, and
$v_{t}^{N}=d_{t}\cos\psi_{t}/\Delta t$. Form
$Z_{1:T}=\Phi_{\mathrm{GK}}\left(R_{1:T},X_{1:T}\right)$, append first and second finite differences, and repeat the first observable motion/difference at window boundaries.3Temporal branchPatch the local history and encode temporal dependencies:
$P_{1:M}=\mathrm{Patch}\left(R_{1:T}\right)$,
$F_{T}=E_{T}\left(P_{1:M};\theta_{T}\right)$, and
${\hat{R}}^{\left(T\right)}=\Pi_{T}\left(F_{T}\right)$.4Geometric–kinematic branchEncode the lifted motion sequence with multi-scale temporal convolutions:
compute $U_{d}=\sigma \left(\mathrm{BN}\left({\mathrm{Conv}}_{\kappa ,d}\left(Z_{1:T}\right)\right)\right)$ for d∈{1,2,4,8},
form $F_{G}=\Pi_{G}\left(\left[U_{1},U_{2},U_{4},U_{8}\right]\right)$, and predict
${\hat{R}}^{\left(G\right)}=\Pi_{G}^{o}\left(F_{G}\right)$.5Branch calibrationExchange complementary temporal and geometric–kinematic information:
${\bar{F}}_{T}=\mathrm{LN}\left(F_{T}+\alpha_{T}\mathrm{Attn}\left(F_{T},F_{G},F_{G}\right)\right)$,
${\bar{F}}_{G}=\mathrm{LN}\left(F_{G}+\alpha_{G}\mathrm{Attn}\left(F_{G},F_{T},F_{T}\right)\right)$.6Branch weight inferenceFor each horizon step, combine the calibrated branch features, their cosine similarity, feature-space $\ell_{2}$ disagreement, and horizon embedding into $q_{h}$. Apply an MLP and horizon-wise self-attention, then infer
${\tilde{G}}_{h}=\sigma \left(w_{2}^{⊤}{\bar{u}}_{h}+b_{2}\right)$ and
$G_{h}={\mathrm{clip}}_{\left[0.1,0.9\right]}\left(a{\tilde{G}}_{h}+b_{h}\right)$.7Fusion, decoding, and trainingGenerate the local prediction by branch-weighted residual correction,
${\hat{r}}_{T+h}={\hat{r}}_{T+h}^{\left(T\right)}+G_{h}⊙\left({\hat{r}}_{T+h}^{\left(G\right)}-{\hat{r}}_{T+h}^{\left(T\right)}\right)$;
decode ${\hat{y}}_{T+h}=\Gamma_{a}^{-1}\left({\hat{r}}_{T+h}\right)$ and optimize the physical MDE plus the 0.1-weighted geometry-branch auxiliary loss in Equation (15).


## 4. Data Acquisition and Preprocessing

The multi-route dataset used in this study was constructed from the OpenSky Network historical database [57]. Flight occurrences and source-provided airport labels were queried from flights_data4. Associated state vectors were retrieved from state_vectors_data4 in blocks no longer than six hours. The verified query interval extended from 5 October 2023 05:02:54 to 25 January 2024 07:52:01 Coordinated Universal Time (UTC). After quality control, the retained trajectories spanned 5 October 2023 05:17:54 to 25 January 2024 07:18:34 UTC. Airport labels were retained only as source metadata because they were not independently verified.

To improve temporal consistency and physical plausibility, the state vectors were aligned to a uniform 5 s reference interval and screened for duplicate, stale, invalid, or anomalous reports. Only short gaps within continuous flight segments were interpolated, and trajectories with insufficient coverage or implausible motion were rejected. Routes affected by ambiguous coordinate wrapping or known OpenSky outages were also excluded. The complete filtering rules and thresholds are provided in the public preprocessing scripts.

The resulting 5 s reference dataset comprised 741,286 position reports from 945 flights. Deterministic downsampling produced matched 10 s and 15 s variants containing 370,879 and 247,407 reports, respectively, from the same flights. The 15 s variant was used in the main experiments, whereas all three variants were evaluated in the sampling-interval sensitivity analysis. The ten routes span Asia, Europe, North America, and Australia over an October–January acquisition period, broadening geographic and temporal coverage beyond the original single-route evaluation. International Civil Aviation Organization 24-bit (ICAO24) identifiers were retained, but aircraft type was not analyzed because no independently verified type field was available.

Flights were partitioned chronologically before sliding-window construction. Training data preceded 6 January 2024 00:00:00 UTC. Validation data extended from that time to immediately before 16 January 2024 00:00:00 UTC, when testing began. The split contained 766 training, 94 validation, and 85 test flights, with every route represented in each partition. Each flight contributed windows to one partition only, preventing flight-level overlap. Each sample contained one aircraft trajectory, so the task was single-trajectory forecasting rather than simultaneous multi-aircraft interaction prediction. Table 1 summarizes the route-level composition of the 15 s dataset used in the main experiments.

## 5. Experimental Setup

All models use the task definition and chronological partitions in Table 2. Models in the common forecasting framework are trained with Adam for at most 100 epochs (early-stopping patience 30; batch size 512); the learning rate is $3.5\times 10^{-4}$ for PatchTST and GeoATF and $1\times 10^{-4}$ for the other neural baselines. FlightBERT++ uses batch size 2048 and its official encoding objective. For models operating on continuous coordinates, coordinate standardizers are fitted only on the training split; FlightBERT++ instead retains its official binary-encoding procedure. One checkpoint per model is selected by validation MDE under seed 2021. The main experiment uses a five-sample (75 s) window displacement. The recurrent baselines use long short-term memory (LSTM) and bidirectional long short-term memory (BiLSTM) architectures. Table 3 therefore reports only architecture-specific hyperparameters.

For protocol consistency, the recurrent baselines encode the true temporal axis and predict local residuals anchored at the last observed position. For the encoder–decoder Transformer, the decoder prefix is formed exclusively from the final observed history points rather than future target labels, thereby preventing target leakage. These safeguards are applied before validation-based checkpoint selection, and no checkpoint in the reported comparison is selected using test-set performance.

### 5.1. Baselines and Metrics

The proposed method is compared with representative trajectory and time-series prediction baselines:

- LSTM and BiLSTM, which provide recurrent neural network baselines.
- Transformer and iTransformer, which represent attention-based sequence modeling.
- PatchTST, which is a strong patch-based time-series forecasting baseline.
- FlightBERT++, which represents binary-encoding-based flight trajectory prediction.

Coordinate-wise errors are measured by mean absolute error (MAE) and root mean square error (RMSE). For a coordinate dimension q∈{λ,ϕ,h}, these metrics are defined as

(16) $$\begin{array}{c} {\mathrm{MAE}}_{q}=\frac{1}{NH}\sum_{i=1}^{N}\sum_{t=1}^{H}\left|y_{i,t}^{q}-{\hat{y}}_{i,t}^{q}\right|,\\ {\mathrm{RMSE}}_{q}=\sqrt{\frac{1}{NH}\sum_{i=1}^{N}\sum_{t=1}^{H}{\left(y_{i,t}^{q}-{\hat{y}}_{i,t}^{q}\right)}^{2}}. \end{array}$$

In addition to coordinate-wise metrics, this paper reports a three-dimensional mean distance error, denoted as MDE, to summarize spatial deviation in kilometers. For evaluation only, the predicted and true WGS-84 geodetic positions are mapped into Earth-Centered, Earth-Fixed (ECEF) coordinates so that longitude, latitude, and altitude deviations can be measured in a common three-dimensional Cartesian frame. For a geodetic point (λ,ϕ,h), the ECEF mapping is

(17) $$\begin{array}{c} N_{\phi }=\frac{a}{\sqrt{1-e^{2}\sin^{2}\phi }},\\ \mathrm{ECEF}\left(\lambda ,\phi ,h\right)=\left[\begin{array}{c} (N_{\phi }+h)\cos\phi \cos\lambda \\ (N_{\phi }+h)\cos\phi \sin\lambda \\ (N_{\phi }\left(1-e^{2}\right)+h)\sin\phi \end{array}\right], \end{array}$$

where *a* and *e* are the WGS-84 ellipsoid semi-major axis and eccentricity, respectively, and *h* is expressed in the same length unit as *a* when MDE is computed. The spatial distance error is then defined as

(18) $$\mathrm{MDE}=\frac{1}{NH}\sum_{i=1}^{N}\sum_{t=1}^{H}{∥\mathrm{ECEF}\left(y_{i,t}\right)-\mathrm{ECEF}\left({\hat{y}}_{i,t}\right)∥}_{2}.$$

The coordinate-wise metrics are retained because they reveal which state variable contributes most to the prediction error. Longitude and latitude errors describe horizontal displacement in angular units, while altitude errors describe vertical deviation in meters. Raw observations and final reported predictions are expressed in WGS-84 geodetic coordinates for coordinate-wise evaluation. Internally, GeoATF transforms each input window into last-observation-anchored local geodesic coordinates and decodes its predictions back to WGS-84, whereas the ECEF mapping is used only inside the reported MDE calculation. The following analysis therefore reports MAE, RMSE, and MDE rather than relying on a single aggregate score.

### 5.2. Statistical Analysis

Statistical inference uses the flight, rather than the highly overlapping sliding window, as the independent analysis unit. For every method and horizon, pointwise WGS-84 ECEF distances are first averaged over the relevant forecast prefix and all test windows within each flight, producing one MDE value per test flight. GeoATF and each baseline are then paired on the same 85 flights. The mean paired reduction (baseline minus GeoATF) is accompanied by a 95% confidence interval (CI) from 10,000 paired bootstrap resamples of the flights. A two-sided Wilcoxon signed-rank test evaluates each paired comparison, and Holm correction controls family-wise error across all 24 baseline–horizon comparisons (six baselines at four horizons). This analysis quantifies test-flight variability for the single predeclared training seed; it is not presented as an estimate of training-seed variability.

## 6. Results

The analysis addresses three questions: whether GeoATF improves prediction accuracy beyond flight-to-flight variability, whether the advantage persists across sampling intervals and flight phases on the multi-route dataset, and which components and learned branch behavior explain the improvement at what computational cost.

### 6.1. Overall Prediction Results

Table 4 shows that GeoATF obtains the lowest MDE at every tested forecast horizon. The strongest competing method changes with horizon: PatchTST ranks second for the one-, four-, and eight-step forecasts, whereas FlightBERT++ becomes second at the 16-step horizon. GeoATF remains first across both comparison regimes, so its advantage is not contingent on a single baseline. At the longest horizon, GeoATF obtains an MDE of 1.9373 km compared with 2.2411 km for FlightBERT++. The coordinate-wise results further show that FlightBERT++ is stronger on several altitude measures at longer horizons, whereas the more balanced horizontal and vertical accuracy of GeoATF yields the lowest combined three-dimensional distance.

As Table 4 pools all test windows, Table 5 reports an additional paired analysis using the flight as the independent unit. The paired reduction is defined as the flight-level MDE of the strongest baseline minus that of GeoATF, so a positive value favors GeoATF. At every forecast horizon, the confidence interval remains above zero and the Holm-adjusted *p* value remains below 0.05. All 24 GeoATF–baseline comparisons are likewise significant after Holm correction. The flight-level evidence therefore confirms that the pooled advantage does not arise from treating overlapping windows as independent observations.

### 6.2. Sampling-Interval Sensitivity

To assess sensitivity to ADS-B reporting frequency, Table 6 compares the three strongest methods under matched input and forecast durations.

Under this matched-duration protocol, the 15 s condition uses a four-sample (60 s) stride and its corresponding validation-selected checkpoint. GeoATF improves monotonically as reporting becomes denser and obtains its lowest errors at 5 s across all physical forecast horizons. PatchTST is also best at 5 s but varies slightly non-monotonically between 10 s and 15 s, whereas FlightBERT++ favors 5 s only for the 30 s forecast and 15 s for the 60–240 s forecasts. These contrasting trends show that sampling sensitivity depends on the model representation rather than producing an identical effect across architectures. GeoATF remains the best of the three methods at every matched interval and physical horizon, although its gradual degradation with coarser sampling shows that it is stable rather than invariant to reduced reporting frequency. These experiments use uniformly resampled sequences and do not establish robustness to irregular message arrival or burst loss.

### 6.3. Ablation Study

To examine the contribution of the core modules, two ablated variants are evaluated. The first removes the geometric–kinematic feature extraction branch and replaces it with a simplified two-layer LSTM. The second retains the geometric–kinematic branch but replaces the adaptive branch-weight fusion module with a simplified gating fusion. Table 7 shows the results.

The ablations separate the roles of the two proposed components. Removing GKFE produces the larger degradation, and the gap widens toward the longer forecast horizons, showing that explicit distance, bearing, and motion-change cues are particularly important when spatial errors accumulate. Replacing ABF causes a smaller but consistent MDE increase because the geometric branch remains available but can no longer adapt its contribution to branch consistency and forecast horizon. The two modules therefore play complementary roles: GKFE supplies the main long-horizon geometric correction, while ABF calibrates how strongly that correction is used. The few short-horizon coordinate-wise exceptions show that the method improves overall three-dimensional multi-step accuracy rather than every individual coordinate in isolation.

### 6.4. Flight-Phase-Specific Analysis

Aircraft trajectory dynamics vary substantially across flight phases. Therefore, the test trajectories are segmented into takeoff/climb, cruise, and descent/landing phases. Table 8 reports the phase-specific three-dimensional mean distance error at each forecast horizon, with the three phases arranged as parallel columns for direct comparison.

Table 8 shows that cruise consistently yields the lowest MDE across methods and horizons. At the longest horizon, the more difficult non-cruise phase depends on the model: PatchTST has its largest error during takeoff/climb, whereas FlightBERT++ and GeoATF have their largest errors during descent/landing. GeoATF obtains the lowest MDE at every forecast horizon during takeoff/climb and cruise and through the eight-step horizon during descent/landing. FlightBERT++ is lower only for the 16-step descent/landing forecast, showing that flight phase and forecast duration jointly affect the model ranking and identifying long-horizon descent/landing as a remaining boundary.

**Figure 4:**
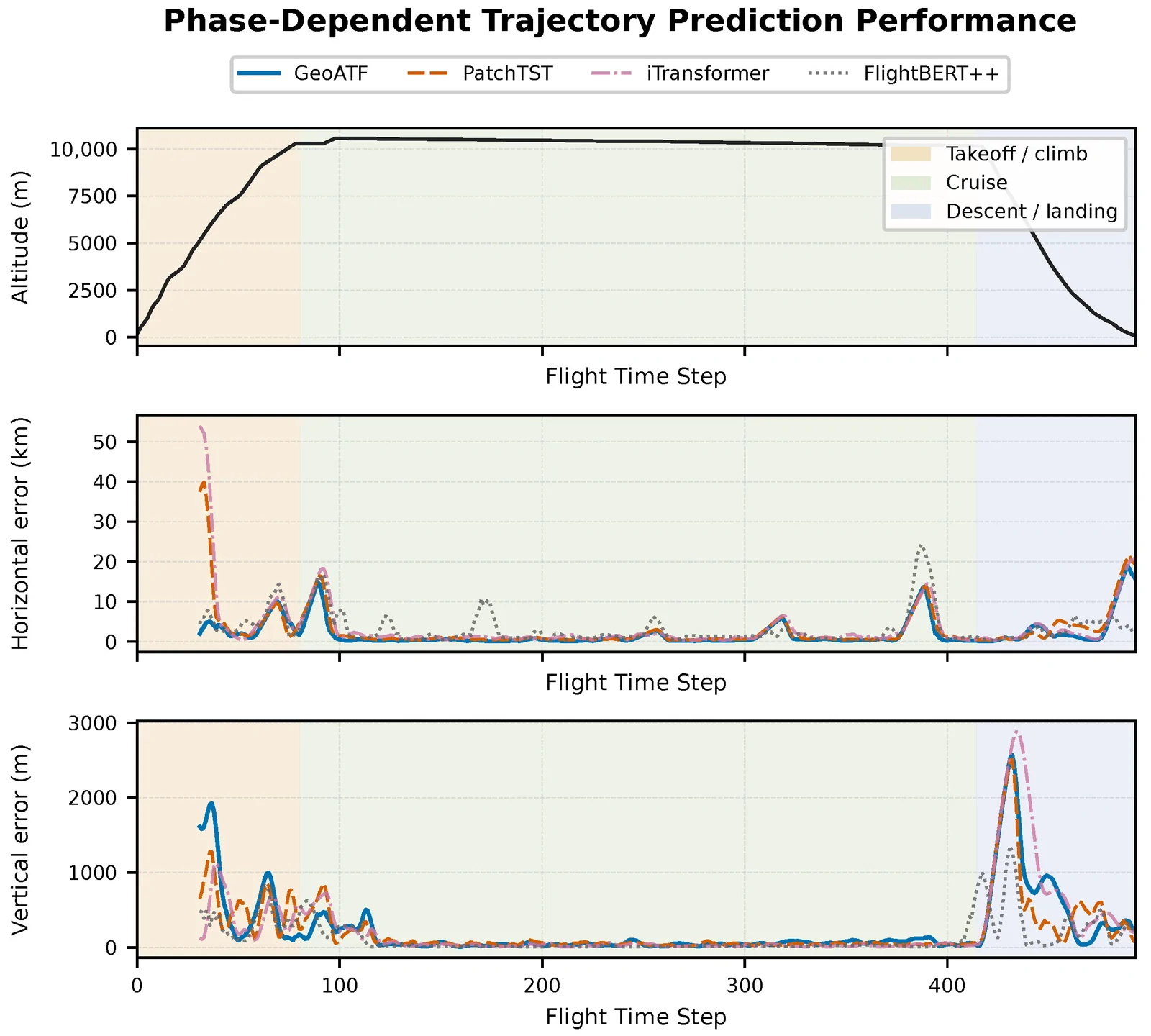
Phase-dependent 16-step prediction for a selected complete flight. The top panel shows altitude and flight phases; the lower panels show horizontal and vertical endpoint errors from stride-one inference, smoothed with a centered five-window moving average for visualization only. Aggregate results are reported in Table 8.

**Figure 5:**
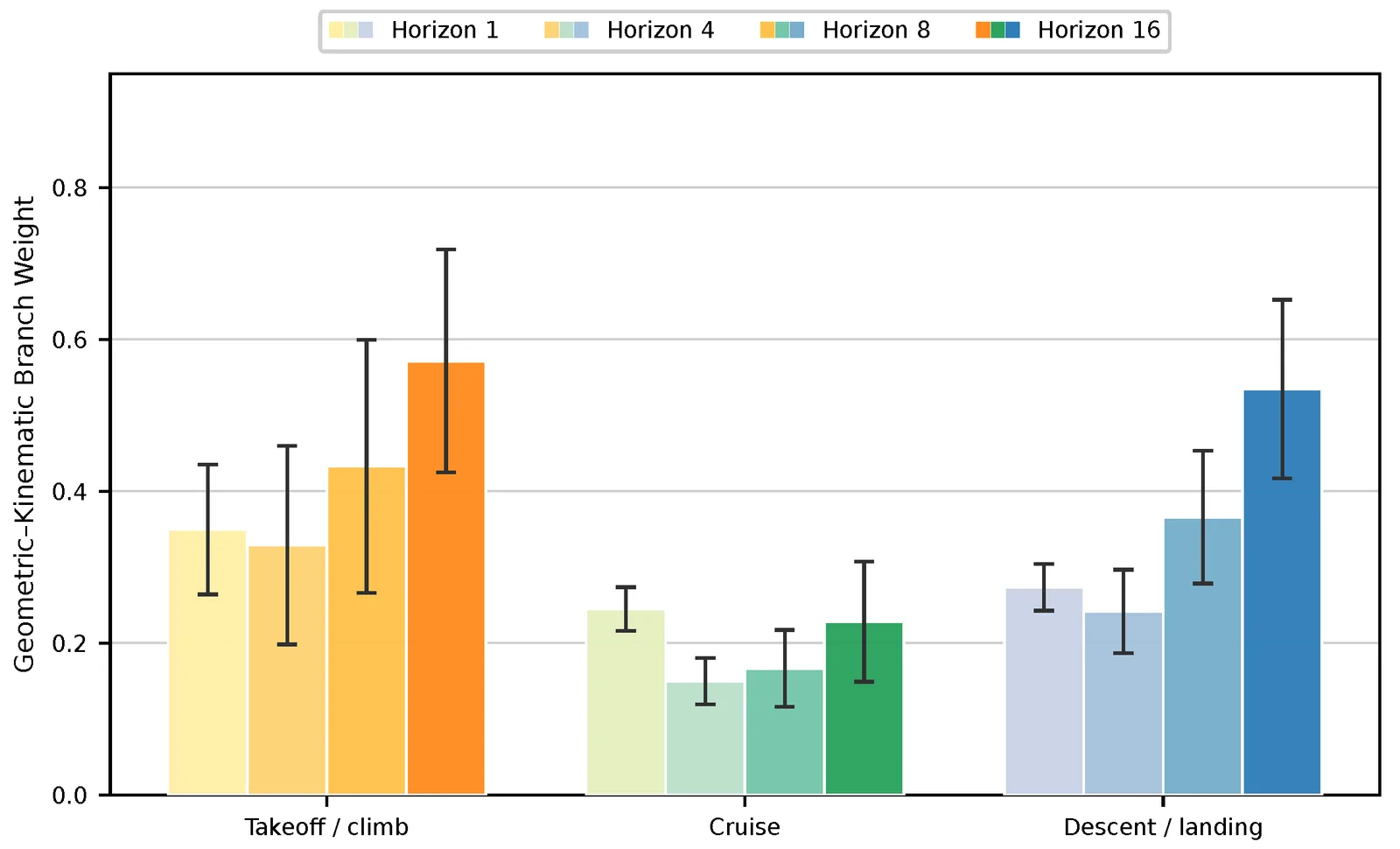
Phase- and horizon-dependent geometric–kinematic branch weights. Bars and error bars denote the across-flight mean and one sample standard deviation after within-flight averaging; higher values indicate a larger geometric–kinematic contribution.

Figure 4 complements the phase table with one selected complete-flight example satisfying the stated coverage criteria. The retained DLH882 flight contains all three phases and is evaluated with the same validation-selected checkpoints; stride-one inference and a five-window moving average are used only to obtain a readable trace, while aggregate results retain the formal stride and unsmoothed errors. GeoATF follows the horizontal and three-dimensional trajectory most closely in this example, although it does not achieve the lowest vertical error. Figure 5 then summarizes the learned geometric–kinematic branch weight after within-flight averaging, with the error bars showing across-flight dispersion.

### 6.5. Qualitative Trajectory Visualization

**Figure 6:**
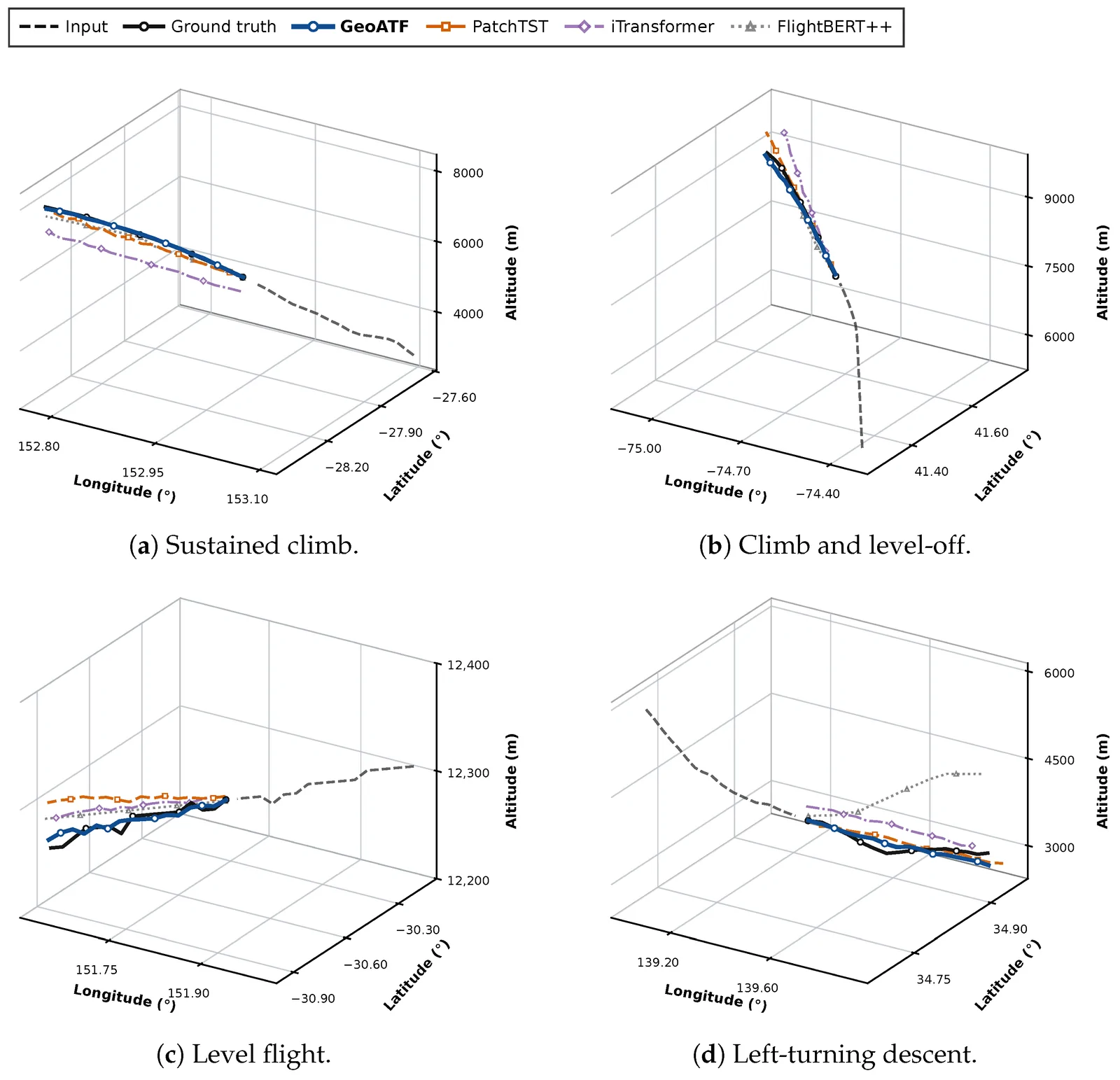
Selected 16-step trajectory forecasts in four future-segment motion regimes. Panels compare the input, ground truth, GeoATF, PatchTST, iTransformer, and FlightBERT++; motion labels use the future ground-truth segment. Aggregate comparisons are reported in Table 4.

Figure 6 presents four selected 16-step forecast examples from distinct future-segment motion regimes. The cases follow documented visualization criteria, and their window identifiers and panel-level metrics are archived with the figure source data. All panels use the retained validation-selected checkpoints without clipping, rescaling, or post-hoc trajectory modification. GeoATF, PatchTST, iTransformer, and FlightBERT++ are distinguished jointly by color, line style, and marker shape so that the comparison remains readable in color and grayscale. These panels illustrate trajectory shape behavior and do not replace the aggregate or flight-level statistical results.

Across the four selected motion regimes, GeoATF preserves the overall trajectory shape while remaining more closely aligned with the ground truth. The competing forecasts remain physically plausible but exhibit larger lateral and/or vertical offsets. The qualitative advantage therefore reflects closer spatial alignment rather than merely the avoidance of divergent failures; the pooled, phase-level, and paired statistics remain the basis for the overall performance claim.

### 6.6. Computational Resource Footprint

Table 9 compares parameter count and additional forward-pass memory under one common 16-step inference protocol (float32, batch size 128, the same graphics processing unit (GPU), and 20 warm-up passes). The memory increment is measured above the warmed model-and-input baseline and therefore characterizes model-forward activation/workspace demand rather than end-to-end deployment memory.

Although GeoATF approximately doubles the parameter count of its PatchTST temporal backbone, their additional forward-memory demands are nearly identical, while GeoATF remains far smaller and lighter than FlightBERT++. The accuracy gain therefore requires a moderate increase in model size but little additional activation/workspace memory relative to PatchTST. This comparison concerns the model forward pass only; acquisition, preprocessing, transfer, storage, and complete streaming latency are outside its scope.

## 7. Discussion

The results support a specific interpretation: representing each window in a last-observation-anchored geodesic chart removes route-dependent absolute offsets, while GKFE supplies distance, bearing, and motion-change information that is absent from generic coordinate regression. This combination is consistent with the larger absolute error reductions observed at longer forecast horizons, where spatial deviations accumulate. The ablation evidence separates the two effects: at the 16-step horizon, removing GKFE raises MDE from 1.9373 to 2.6587 km, whereas replacing adaptive branch weighting raises it to 2.0097 km. The paired flight-level analysis further shows that the short-horizon improvement is not an artifact of counting overlapping windows as independent samples.

The phase results refine this interpretation instead of repeating the aggregate ranking. Cruise generally exhibits smoother altitude evolution and greater lateral consistency and receives a lower mean geometry-branch weight. Takeoff/climb and descent/landing show larger and more dispersed weights, indicating greater sample-level variation in the useful geometric correction. GeoATF retains the lowest phase-level MDE through the eight-step horizon and at the 16-step horizon for takeoff/climb and cruise. During 16-step descent/landing prediction, however, FlightBERT++ attains 3.0436 km compared with 3.2710 km for GeoATF. This phase- and horizon-specific exception qualifies the pooled ranking and identifies descent/landing at the longest horizon as a remaining limitation.

The evidence supports short-term forecasting under the evaluated protocol, not immediate operational deployment. The ten-route dataset is substantially broader than the original single-route experiment; however, all three chronological splits contain the same route identities. The experiment therefore measures temporal generalization to later flights on those routes, not leave-one-route-out, unseen-region, or independent-dataset generalization. Uniform 5, 10, and 15 s resampling also does not reproduce arbitrary message loss or irregular arrival. Moreover, the model uses only historical longitude, latitude, and altitude and omits weather, flight plans, aircraft intent and type, airspace restrictions, standard departure/arrival procedures, and controller instructions.

These limitations define the next validation steps. Future work should test leave-one-route-out and cross-dataset transfer, retain verified aircraft-type metadata, model irregular or missing ADS-B reports, and integrate weather and operational intent through controlled ablations. Uncertainty-aware forecasts and complete streaming measurements—including acquisition, preprocessing, transfer, and end-to-end latency—are also required before safety-related or real-time feasibility claims can be made. The present resource comparison is limited to model parameters and peak forward-memory increment under a common batch protocol.

## 8. Conclusions

This paper presented GeoATF, a geometry-informed adaptive time-series fusion framework for short-term aircraft trajectory prediction from ADS-B position reports. The revised implementation predicts local up–east–north offsets in a last-observation-anchored geodesic chart, derives physical great-circle and kinematic descriptors from the observed WGS-84 positions, and adaptively fuses temporal and geometric–kinematic branch predictions under a physical MDE objective.

On 945 flights from ten routes, GeoATF achieved the lowest pooled MDE at all four horizons and a 1.9373 km MDE at 240 s, 13.6% below the strongest baseline. All 24 paired flight-level comparisons remained significant after Holm correction, and the sampling, ablation, phase, qualitative, and resource analyses identified both the benefits and the operating boundaries of the method. These findings apply to later flights from the evaluated route set under uniformly resampled ADS-B observations. They do not establish unseen route, irregular message, or operational system performance. Future work will prioritize independent route and independent dataset validation, richer weather and intent inputs, verified aircraft metadata, missing-message robustness, and uncertainty-aware prediction.

## Figures and Tables

**Table 1:** Route-level composition of the 15 s dataset used in the main experiments. Flights were split chronologically before sliding-window construction. Bold values denote aggregate totals.

Flight ID	Route	Train	Validation	Test	Flights	Position Reports
ANA992	RJBB–RJTT	76	10	7	93	21,284
BAW430	EGLL–EHAM	76	10	8	94	17,141
DLH882	EDDF–EETN	68	9	5	82	39,125
JBU1318	KJFK–KBOS	79	9	10	98	15,335
JST503	YSSY–YMML	75	8	9	92	26,799
QFA501	YBBN–YSSY	84	10	9	103	30,974
RPA4308	KLGA–CYYZ	86	10	9	105	27,660
SIA103	WMKK–WSSS	64	8	10	82	13,507
SWR511P	LFPG–LSZH	82	10	9	101	19,610
UAL1888	KATL–KORD	76	10	9	95	35,972
**Total**		**766**	**94**	**85**	**945**	**247,407**

**Table 2:** Experimental configuration used for the Automatic Dependent Surveillance-Broadcast (ADS-B)-based trajectory prediction task.

Item	Setting
Input variables	Longitude, latitude, altitude
Coordinate representation	Raw and reported states in World Geodetic System 1984 (WGS-84) longitude–latitude–altitude; GeoATF predicts normalized anchor-relative up–east–north offsets and decodes them by great-circle forward navigation
Dataset scope	945 flights on ten routes; 5 October 2023–25 January 2024 UTC
Sampling interval	15 s
Historical window	16 samples (240 s)
Prediction horizons	1, 4, 8, and 16 samples (15, 60, 120, and 240 s, respectively)
Maximum prediction time	240 s
Dataset split	766 training, 94 validation, and 85 test flights under the chronological cutoffs specified in Section 4
Implementation	PyTorch 2.9.0 with CUDA 13.0
Hardware	NVIDIA RTX PRO 6000 Blackwell Server Edition GPU (NVIDIA Corporation, Santa Clara, CA, USA)

**Table 3:** Model-specific hyperparameters used in the reported experiments.

Model	Architecture-Specific Hyperparameters
LSTM	2 recurrent layers; hidden dimension 128
BiLSTM	2 bidirectional recurrent layers; hidden dimension 128
Transformer	$d_{\mathrm{model}}=128$; $d_{\mathrm{ff}}=256$; 8 attention heads; 3 encoder layers; 1 decoder layer
iTransformer	$d_{\mathrm{model}}=128$; $d_{\mathrm{ff}}=256$; 8 attention heads; 3 encoder layers
PatchTST	Patch length 8; stride 2; $d_{\mathrm{model}}=128$; $d_{\mathrm{ff}}=256$; 32 attention heads; 3 encoder layers; reversible instance normalization (RevIN) enabled
FlightBERT++	$n_{\mathrm{embd}}=768$; 4 attention heads; 4 encoder layers; 2 decoder layers; dropout 0.1; Gray coding disabled
GeoATF	PatchTST branch as above; GKFE hidden dimension 128 with dilation rates {1,2,4,8}; ABF hidden dimension 64 with 8 attention heads

**Table 4:** Comprehensive performance comparison on the ADS-B-based flight trajectory prediction task. Lower values are better. Bold indicates the best result.

Method	Horizon	MAE ↓				RMSE ↓				MDE (km) ↓
		Lon	Lat	Alt		Lon	Lat	Alt		
LSTM	1	0.0081	0.0057	65.88		0.0119	0.0082	93.50		1.0737
	4	0.0202	0.0141	161.95		0.0323	0.0224	255.25		2.6741
	8	0.0362	0.0253	287.35		0.0592	0.0412	460.74		4.7970
	16	0.0684	0.0475	530.45		0.1125	0.0779	839.98		9.0075
BiLSTM	1	0.0050	0.0033	66.82		0.0076	0.0047	94.02		0.6282
	4	0.0122	0.0080	165.32		0.0202	0.0124	255.55		1.5337
	8	0.0214	0.0141	294.87		0.0363	0.0223	459.63		2.6983
	16	0.0394	0.0256	543.81		0.0679	0.0407	834.93		4.9422
Transformer	1	0.0232	0.0154	250.51		0.0315	0.0201	391.51		2.8221
	4	0.0217	0.0145	296.41		0.0305	0.0192	457.81		2.6936
	8	0.0265	0.0176	383.87		0.0382	0.0247	593.29		3.2716
	16	0.0432	0.0302	557.77		0.0661	0.0459	858.81		5.4816
iTransformer	1	0.0043	0.0031	105.83		0.0061	0.0044	170.72		0.5834
	4	0.0065	0.0050	137.94		0.0119	0.0092	225.18		0.9020
	8	0.0112	0.0087	183.11		0.0246	0.0184	306.81		1.5408
	16	0.0244	0.0185	288.05		0.0564	0.0406	500.03		3.2801
PatchTST	1	0.0014	0.0012	27.02		0.0023	0.0018	43.88		0.2014
	4	0.0036	0.0025	54.57		0.0080	0.0056	98.23		0.4681
	8	0.0080	0.0056	101.64		0.0200	0.0137	191.27		1.0340
	16	0.0215	0.0149	221.14		0.0523	0.0350	414.76		2.7335
FlightBERT++	1	0.0017	0.0013	23.43		0.0034	0.0024	39.95		0.2442
	4	0.0041	0.0031	54.06		0.0088	0.0064	102.45		0.5711
	8	0.0080	0.0060	**94.53**		0.0172	0.0126	**180.16**		1.0977
	16	0.0164	0.0125	**168.39**		0.0345	**0.0254**	**311.14**		2.2411
GeoATF	1	**0.0012**	**0.0009**	**21.84**		**0.0019**	**0.0015**	**34.74**		**0.1591**
	4	**0.0027**	**0.0021**	**51.53**		**0.0061**	**0.0045**	**95.31**		**0.3742**
	8	**0.0059**	**0.0044**	99.80		**0.0142**	**0.0105**	189.21		**0.7982**
	16	**0.0142**	**0.0109**	218.22		**0.0336**	0.0257	406.95		**1.9373**

**Table 5:** Flight-level paired statistical comparison between GeoATF and the strongest baseline at each forecast horizon. MDE and paired reductions are reported in kilometers.

Horizon	Strongest Baseline	GeoATF MDE	Baseline MDE	Paired Reduction (95% CI)	Holm-Adjusted *p*
1	PatchTST	0.1666	0.2060	0.0395 (0.0315–0.0471)	$7.92\times 10^{-12}$
4	PatchTST	0.4052	0.5007	0.0955 (0.0800–0.1105)	$5.37\times 10^{-13}$
8	PatchTST	0.8868	1.1293	0.2426 (0.2063–0.2780)	$2.56\times 10^{-13}$
16	FlightBERT++	2.1744	2.4172	0.2427 (0.1289–0.3645)	$4.39\times 10^{-5}$

**Table 6:** Sampling-rate sensitivity in MDE (km) under matched 240 s input/output duration and 60 s sliding-window displacement. Lower values are better. Bold indicates the minimum within each method at each physical forecast horizon.

Method	Interval	30 s	60 s	120 s	180 s	240 s
GeoATF	5 s	**0.1905**	**0.3248**	**0.7085**	**1.1992**	**1.7742**
	10 s	0.2063	0.3527	0.7449	1.2361	1.8073
	15 s	0.2409	0.3943	0.8038	1.3121	1.9023
PatchTST	5 s	**0.2199**	**0.3913**	**0.9110**	**1.6368**	**2.5283**
	10 s	0.3021	0.4711	1.0209	1.7833	2.7095
	15 s	0.2676	0.4630	1.0255	1.7870	2.7097
FlightBERT++	5 s	**0.3273**	0.5885	1.1673	1.7709	2.3777
	10 s	0.3308	0.5761	1.1199	1.6839	2.2647
	15 s	0.3428	**0.5669**	**1.0732**	**1.6234**	**2.1933**

**Table 7:** Ablation study on the ADS-B-based flight trajectory prediction task. Lower values are better. Bold indicates the best result.

Method	Horizon	MAE ↓				RMSE ↓				MDE (km) ↓
		Lon	Lat	Alt		Lon	Lat	Alt		
PatchTST	1	0.0014	0.0012	27.02		0.0023	0.0018	43.88		0.2014
	4	0.0036	0.0025	54.57		0.0080	0.0056	98.23		0.4681
	8	0.0080	0.0056	101.64		0.0200	0.0137	191.27		1.0340
	16	0.0215	0.0149	221.14		0.0523	0.0350	414.76		2.7335
w/o GKFE	1	0.0015	0.0011	**20.22**		0.0024	0.0017	**32.95**		0.1921
	4	0.0033	0.0024	**50.84**		0.0076	0.0054	99.88		0.4315
	8	0.0076	0.0054	104.20		0.0192	0.0133	209.57		0.9860
	16	0.0208	0.0145	239.41		0.0506	0.0342	463.84		2.6587
w/o ABF	1	**0.0012**	0.0011	25.25		0.0021	0.0017	37.85		0.1803
	4	0.0030	0.0025	62.49		0.0062	0.0049	104.35		0.4257
	8	0.0062	0.0049	114.36		**0.0142**	0.0107	195.50		0.8560
	16	0.0147	0.0115	227.66		0.0341	**0.0257**	**389.42**		2.0097
GeoATF	1	**0.0012**	**0.0009**	21.84		**0.0019**	**0.0015**	34.74		**0.1591**
	4	**0.0027**	**0.0021**	51.53		**0.0061**	**0.0045**	**95.31**		**0.3742**
	8	**0.0059**	**0.0044**	**99.80**		**0.0142**	**0.0105**	**189.21**		**0.7982**
	16	**0.0142**	**0.0109**	**218.22**		**0.0336**	**0.0257**	406.95		**1.9373**

**Table 8:** Flight-phase-specific mean distance error (MDE, km) at four forecast horizons; lower values are better. Bold indicates the best result.

Method	Horizon	Takeoff/Climb	Cruise	Descent/Landing
PatchTST	1	0.3568	0.1712	0.2290
	4	0.8611	0.3301	0.7069
	8	2.0988	0.6438	1.7318
	16	5.6716	1.6644	4.7281
FlightBERT++	1	0.3626	0.2172	0.2846
	4	0.8986	0.4688	0.7568
	8	1.6761	0.8981	1.5122
	16	2.8762	1.9050	**3.0436**
GeoATF	1	**0.2958**	**0.1252**	**0.2046**
	4	**0.6563**	**0.2602**	**0.5898**
	8	**1.2549**	**0.5425**	**1.3536**
	16	**2.4722**	**1.3904**	3.2710

**Table 9:** Model size and peak forward-memory increment under the common 16-step inference protocol; lower values are better. Bold indicates the best result.

Method	Parameters (M) ↓	Memory Increment (MB) ↓
LSTM	**0.206**	40.30
BiLSTM	0.211	53.50
Transformer	0.617	14.62
iTransformer	0.402	**4.65**
PatchTST	0.461	15.28
FlightBERT++	34.709	124.38
GeoATF	0.900	15.35

## Data Availability

The source ADS-B observations were obtained from the OpenSky Network historical database, for which access is governed by OpenSky account and data-use conditions. Section 4 specifies the source tables, exact UTC acquisition interval, routes, temporal query blocks, filtering criteria, resampling procedure, and chronological split boundaries required to reconstruct the sample. The query and preprocessing scripts, route definitions, model implementations, and experiment scripts are publicly available at https://github.com/wyfeng53/GET-NETv2 (accessed on 2 August 2026). Processed trajectory files and trained weights are available from the corresponding author upon reasonable request, subject to OpenSky redistribution terms and institutional data-use conditions.

## Abbreviations

The following abbreviations are used in this manuscript:

ABF	Adaptive branch-weight fusion
ADS-B	Automatic Dependent Surveillance-Broadcast
ATM	Air traffic management
ECEF	Earth-Centered, Earth-Fixed
GKFE	Geometric–kinematic feature extraction
MAE	Mean absolute error
MDE	Mean distance error
RevIN	Reversible instance normalization
RMSE	Root mean square error
SAR	Synthetic aperture radar
TBO	Trajectory-based operations
